# Synthesis and Antimicrobial Activity of Silver-Doped Hydroxyapatite Nanoparticles

**DOI:** 10.1155/2013/916218

**Published:** 2012-12-30

**Authors:** Carmen Steluta Ciobanu, Simona Liliana Iconaru, Mariana Carmen Chifiriuc, Adrian Costescu, Philippe Le Coustumer, Daniela Predoi

**Affiliations:** ^1^Department of Multifunctional Materials and Structures Laboratory, National Institute of Materials Physics, 105 Bis Atomistilor, P.O. Box MG 07, 077125 Magurele, Romania; ^2^Faculty of Physics, University of Bucharest, 405 Atomistilor, CP MG-1, 077125 Magurele, Romania; ^3^Microbiology Department, Faculty of Biology, University of Bucharest, Aleea Portocalelor 1-3, 60101 Bucharest, Romania; ^4^EA 4592 Géoressources & Environnement, EGID, Universite Bordeaux, 1 Allée F. Daguin 18, 33607 Pessac Cedex, France

## Abstract

The synthesis of nanosized particles of Ag-doped hydroxyapatite with antibacterial properties is of great interest for the development of new biomedical applications. The aim of this study was the evaluation of Ca_10−*x*_Ag_*x*_(PO_4_)_6_(OH)_2_ nanoparticles (Ag:HAp-NPs) for their antibacterial and antifungal activity. Resistance to antimicrobial agents by pathogenic bacteria has emerged in the recent years and became a major health problem. Here, we report a method for synthesizing Ag doped nanocrystalline hydroxyapatite. A silver-doped nanocrystalline hydroxyapatite was synthesized at 100°C in deionised water. Also, in this paper Ag:HAp-NPs are evaluated for their antimicrobial activity against Gram-positive and Gram-negative bacteria and fungal strains. The specific antimicrobial activity revealed by the qualitative assay is demonstrating that our compounds are interacting differently with the microbial targets, probably due to the differences in the microbial wall structures.

## 1. Introduction 

In the last years nanotechnology and engineered nanoparticles have become an emerging field in the area of materials science. Nanotechnology manipulates matter at an atomic scale creating new nanoproducts with novel properties [1, 2]. The novel properties of those types of nanoparticles have been widely investigated for their use in medicine [3] cosmetics [4], environment [5], and technology. One of the most challenging fields of materials science is the one involving biomaterials. The scientific research takes a lot of effort in order to provide new and improved biomaterials with specific applications in medicine [6, 7]. The continuing appearance of antibiotic resistance in microorganisms challenges the scientific community to constantly develop new bioactive compounds and drug targets with high biocompatibility and antibacterial properties [8–10]. Nowadays, the scientists are looking towards developing new bioactive compounds with silver at a nanometric scale. Although the exact mechanism of the antibacterial action of Ag nanoparticles is not completely understood, there are reports in the literature that show that electrostatic attraction between negatively charged bacterial cells and positively charged nanoparticles is crucial for the activity of nanoparticles as bactericidal materials [11, 12]. Several possible mechanisms have been proposed that involve the interaction of silver with biological macromolecules [13] such as enzymes and DNA through an electron-release mechanism [14]. It is believed that DNA loses its replication ability and cellular proteins are inactivated upon Ag^+^ treatment [15]. In addition, it has also shown that Ag^+^ binds to functional groups of proteins, resulting in protein denaturation [16]. In order to obtain high biocompatibility and optimal antibacterial properties of the new compounds, silver ions were embedded in materials with bio-properties [17]. One of the most studied biomaterial for its extraordinary biocompatibility, bioactivity, and osteoconductivity is the hydroxyapatite (HAp), a bioceramic material from the family of apatites with the general formula Ca_10_(PO_4_)_6_(OH)_2_ [18, 19]. Being the main inorganic constituent in human bones and teeth, HAp is widely used in medical applications such as implants, coatings, prostheses. Due to the high demand for new biocompatible and antibacterial materials, the scientists thought of immobilizing antibacterial metals in the matrix of biomaterials. 

The aim of this study was to evaluate Ag:HAp-NPs for their antimicrobial activities against Gram-positive and Gram-negative bacteria and fungal strains. The preparation of Ag doped hydroxyapatite by coprecipitation method at 100°C has several advantages over other techniques [17]. In our previous studies [17] we presented preliminary results regarding the synthesis, characterization, and antibacterial properties on hydroxyapatite (HAp) and silver doped hydroxyapatite (Ag:HAp) with *x*_Ag_ = 0.2. In this study we present complex studies on silver doped hydroxyapatite samples for different silver concentration: 0.2 ≤ *x*_Ag_ ≤ 0.4.

 Specifically, it can generate highly crystalline nanopowder Ag:HAp. The structure, morphology, and optical properties of the obtained samples were systematically characterized by X-ray diffraction (XRD), transmission electron microscopy (TEM), scanning electron microscopy (SEM), and Fourier transform infrared (FT-IR). For revealing the uniform distribution of the silver in the Ag:HAp nanopowder the XPS results are also presented. This work is focused on the results of complex antimicrobial studies conducted on several strains. Also, in this paper we report the minimal inhibitory concentration and biofilm active concentration on the microbial strains susceptible to the tested compounds in the qualitative screening assay. The biological assay results presented in this manuscript are original and the only ones in the literature performed on this type of materials, demonstrating its antimicrobial potential. 

## 2. Materials and Methods

### 2.1. Synthesis of Ag^+^-Doped Hydroxyapatite

All the reagents for synthesis including ammonium dihydrogen phosphate [(NH_4_)_2_HPO_4_], calcium nitrate [Ca(NO_3_)_2_·4H_2_O], and silver nitrate AgNO_3_ were purchased from Alpha Aesare and used without further purification. 

The Ca_10−*x*_Ag_*x*_(PO_4_)_6_(OH)_2_, with *x*_Ag_ = 0.2, *x*_Ag_ = 0.3, and *x*_Ag_ = 0.4, ceramic powder, was prepared (Ca/P molar ratio: 1.67) using Ca(NO_3_)_2_·4H_2_O and (NH_4_)_2_HPO_4_ by coprecipitation. For silver doped hydroxyapatite nanoparticles the ratio [Ca+Ag]/P was 1.67. AgNO_3_ and Ca(NO_3_)_2_·4H_2_O were dissolved in deionised water to a final volume of 300 mL [Ca+Ag]-containing solution. (NH_4_)_2_HPO_4_ was dissolved in deionised water to a final volume of 300 mL P-containing solution. The [Ca+Ag]-containing solution was stirred at 100°C for 30 minutes. The P-containing solution with a pH of 10 (adjusted by NH_3_) was added drop by drop into the [Ca+Ag]-containing solution and stirred for 2 h. The pH value was constantly adjusted at 10 during the reaction. Afterwards, the precipitate was washed several times with deionised water. The resulting material was dried at 100°C for 72 h. 

### 2.2. Sample Characterization

The X-ray diffraction measurements of Ca_10−*x*_Ag_*x*_(PO_4_)_6_(OH)_2_ samples were recorded using a Bruker D8 Advance diffractometer, with nickel filtered CuK_*α*_ (*λ* = 1.5418 Å) radiation, and a high efficiency one-dimensional detector (Lynx Eye type) operated in integration mode. The diffraction patterns were collected in the 2*θ* range 15°–140°, with a step of 0.02° and 34 s measuring time per step. Scanning electron microscopy (SEM) study was performed on a HITACHI S2600N-type microscope equipped with an energy dispersive X-ray attachment (EDAX/2001 device). Energy Dispersive X-Ray Analysis (EDX), referred to as EDS or EDAX, was used to identify the elemental composition of materials. Transmission electron microscopy (TEM) studies were carried out using a FEI Tecnai 12 equipped with a low dose digital camera from Gatan. The specimen for TEM imaging was prepared by ultramicrotomy to get thin section about 60 nm of thickness. The powder is embedded in an epoxy resin (polaron 612) before microtomy. TEM modes used were Bright Field (BF). The functional groups present in the prepared nanoparticles and thin films were identified by FTIR using a Spectrum BX spectrometer. To obtain the nanoparticles spectra 1% of nanopowder was mixed and ground with 99% KBr. Tablets of 10 mm diameter were prepared by pressing the powder mixture at a load of 5 tons for 2 min. The spectrum was taken in the range of 500 to 4000 cm^−1^ with 4 cm^−1^ resolution. X-Ray Photoelectron Spectroscopy (XPS) studies were conducted using a VG ESCA 3 MK II XPS installation (*E*_K*α*_ = 1486.7 eV). The vacuum analysis chamber pressure was *P* ~ 3 × 10^−8^ torr. The XPS recorded spectrum involved an energy window *w* = 20 eV with the resolution *R* = 50 eV with 256 recording channels. The XPS spectra were processed using Spectral Data Processor v 2.3 (SDP) software. 

### 2.3. The *In Vitro* Antibacterial and Antifungal Activity

The antimicrobial activities of the tested substances were determined against ATCC reference and clinical microbial strains, that is, Gram-positive (*Bacillus subtilis*, *Staphylococcus aureus* 0364, *Enterococcus faecalis* ATCC 29212) and Gram-negative (*Escherichia coli* ATCC 25922, *Klebsiella pneumoniae* 2968, *Enterobacter cloacae* 61R, *Pseudomonas aeruginosa* 1397) bacterial and yeast (*Candida krusei* 963) strains. The microbial strains identification was confirmed by aid of VITEK 2 automatic system. VITEK cards for identification and susceptibility testing were inoculated and incubated according to the manufacturer's recommendations. The results were interpreted by using software version AMS R09.1.

Microbial suspensions of 1.5 × 10^8^ CFU/mL corresponding to a 0.5 McFarland density obtained from 15–18 h bacterial cultures developed on solid media were used in our experiments. The antimicrobial activity was tested on Mueller-Hinton Agar (MHA) medium, while a Yeast Peptone Glucose (YPG) medium was used in case of *C. krusei*. The tested substances were solubilised in DMSO and the starting stock solution was of 5000 *μ*g/mL concentration. The qualitative screening was performed by an adapted disk diffusion method [20–24]. The quantitative assay of the antimicrobial activity against planktonic microbial strains was performed by the liquid medium microdilution method, in 96 multi-well plates, in order to establish the minimal inhibitory concentration (MIC). For this purpose, serial two-fold dilutions of the compounds ranging between 5000 and 1.95 *μ*g/mL were performed in a 200 *μ*L volume of broth and each well was seeded with 50 *μ*L microbial inoculum. Sterility control (wells containing only culture medium) and culture controls (wells containing culture medium seeded with the microbial inoculum) were used. The influence of the DMSO solvent was also quantified in a series of wells containing DMSO, diluted accordingly with the dilution scheme used for the complexes. The plates were incubated for 24 hrs at 37°C. The MIC values were considered as the lowest concentration of the tested compound that inhibited the visible growth of the microbial overnight cultures [20–24]. The assessment of the complexes influence on the microbial ability to colonize an inert substratum was performed by the micro-titer method. For this purpose, the microbial strains have been grown in the presence of two-fold serial dilutions of the tested compounds performed in liquid nutrient broth/YPG, distributed in 96-well plates, and incubated for 24 hours at 37°C for bacterial strains, for 48 hours at 28°C, respectively, for fungal strains. At the end of the incubation period, the plastic wells were emptied, washed three times with phosphate buffered saline (PBS), fixed with cold methanol and stained with 1% violet crystal solution for 30 minutes. The biofilm formed on plastic wells was resuspended in 30% acetic acid. The intensity of the coloured suspensions was assessed by measuring the absorbance at 490 nm. The last concentration of the tested compound that inhibited the development of microbial biofilm on the plastic wells was considered the minimum inhibitory concentration of the biofilm development and was also expressed in *μ*g/mL [25–28].

## 3. Results and Discussion

**Figure 1:**
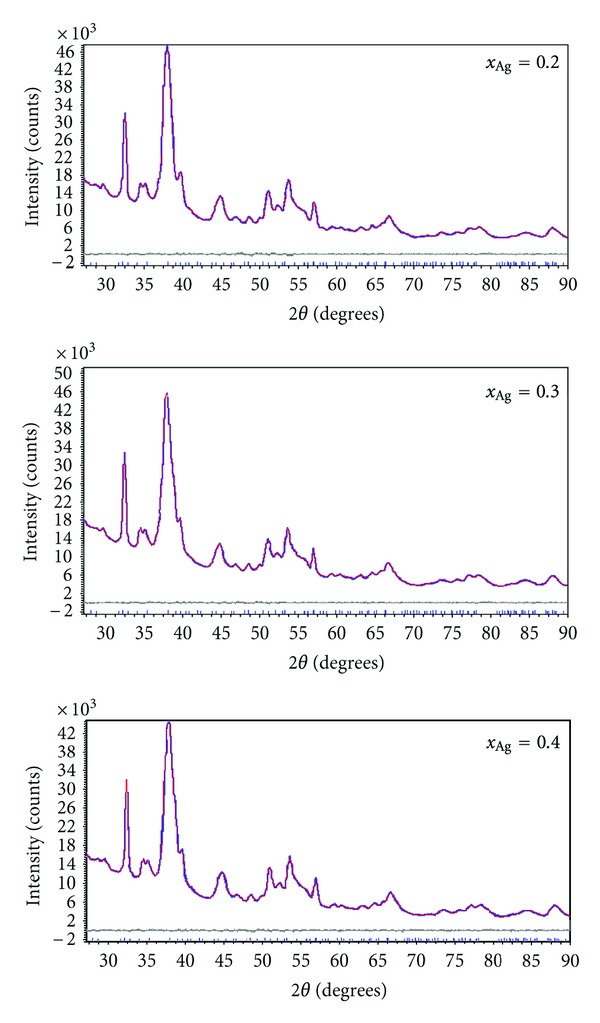
Rietveld refinement plot of the Ag:HAp samples synthesized with *x*_Ag_ = 0.2, *x*_Ag_ = 0.3, and *x*_Ag_ = 0.4. The observed data are represented in blue and the calculated data by a red line. Vertical lines represent the positions of diffraction lines of hydroxyapatite (ICDD-PDF number 9-432). The line below the gray plot is the difference profile.

The designed unit formula of the doped HAp is Ca_10−*x*_Ag_*x*_(PO_4_)_6_(OH)_2_, with 0.2 ≤ *x* ≤ 0.4. The XRD patterns, presented in Figure 1, show the characteristic peaks of hydroxyapatite for each sample, according to ICDD-PDF number 9-432, represented at the bottom of the figure, as reference. No other crystalline phases were detected beside this phase (Figure 1).

We performed whole powder pattern fitting by the Rietveld method of the as-prepared Ag-HAp structures. As a prerequisite for the atomic structure refinement, a good fit of the diffraction line profiles must be achieved. Good pattern fit has been achieved using MAUD (Material Analysis Using Diffraction) [29] for all the samples, by applying the Popa approach for the anisotropic microstructure analysis [30] implemented in the MAUD code as “Popa rules”. It resulted that each sample is constituted of elongated nanocrystallites which can be approximated by circular ellipsoids, with the longer dimension parallel to the *c* crystallographic axis of HAp.

Figure 1 shows the XRD patterns of Ag:HAp (*x*_Ag_ = 0.2, *x*_Ag_ = 0.3 and *x*_Ag_ = 0.4) and the standard data for the hexagonal hydroxyapatite, respectively. In the case of the Ag:HAp doped samples (*x*_Ag_ = 0.2, *x*_Ag_ = 0.3 and *x*_Ag_ = 0.4), the diffraction peaks can be well indexed to the hexagonal Ca_10_(PO_4_)_6_(OH)_2_ in *P*63/*m* space group (ICDD-PDF No. 9-432). On the other hand, the characteristic diffractions of hexagonal HAp are still obvious, and no other phases related with doped component can be detected.

The calculated lattice constants of *a* = *b* = 0.9422 nm, *c* = 0.6879 nm for Ca_10−*x*_Ag_*x*_(PO_4_)_6_(OH)_2_, with *x*_Ag_ = 0.2, *a* = *b* = 0.9423 nm, *c* = 0.6878 nm when *x*_Ag_ = 0.3 and *a* = *b* = 0.9427 nm, *c* = 0.6877 nm for Ca_10−*x*_Ag_*x*_(PO_4_)_6_(OH)_2_, with *x*_Ag_ = 0.4 are in good agreement with the standard data of *a* = *b* = 0.9418 nm, *c* = 0.6884 nm (spacegroup *P*63/*m*). The resulting Bragg *R*-factor and chi squared (*χ* ^2^) were given 0.057 and 1.088 for samples with *x*_Ag_ = 0.4. For the samples with *x*_Ag_ = 0.3 the Bragg *R*-factor and chi squared (*χ* ^2^) were given 0.045 and 1.065 while for samples with *x*_Ag_ = 0.2 the Bragg *R*-factor and chi squared (*χ* ^2^) are 0.059 and 1.071, respectively. The XRD of Ag:HAp also demonstrates that powders made by coprecipitation at 100°C exhibit the apatite characteristics with good crystal structure and no new phase or impurity is found.

**Figure 2:**
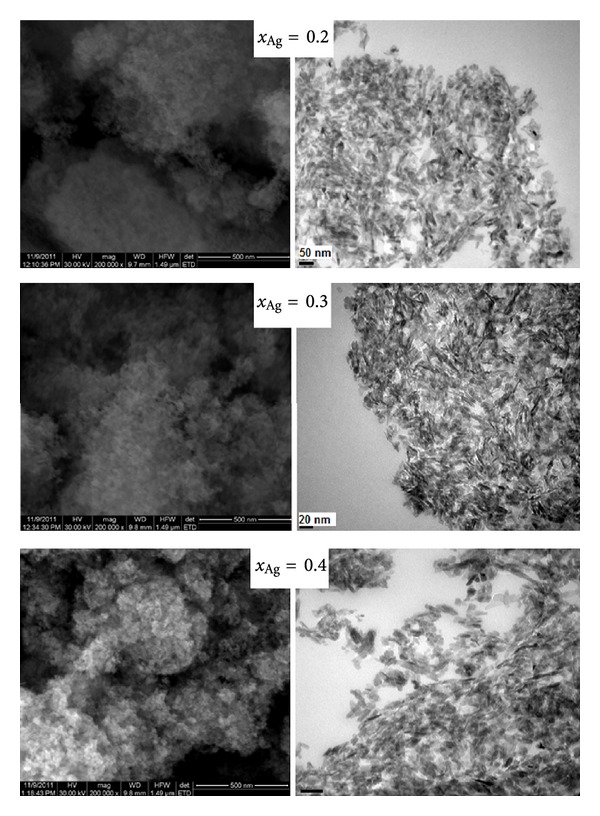
SEM images (left) and TEM micrographies (right) of the Ag:HAp samples with *x*_Ag_ = 0.2, *x*_Ag_ = 0.3, and *x*_Ag_ = 0.4.

The TEM micrographies give information on the texture (Bright Field) and the structure (SAED) of the three samples (*x*_Ag_ = 0.05, 0.2, and 0.3). All the samples exhibit a uniform rod-like morphology with particles from 30 nm to 5 nm, as observed on the BF micrographies (Figure 2). The results suggested that the doping by Ag^+^ have little influence on the size of HAp nanoparticles. The samples prepared at *x*_Ag_ = 0.4 exhibited much smaller particle size. 

**Figure 3:**
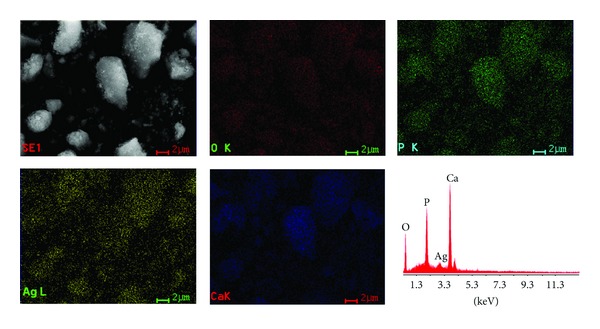
EDAX spectrum of the Ag:HAp samples and simultaneous distributions of individual elements based on selected region of the sample (*x*_Ag_ = 0.4).

SEM images provide direct information about the size and typical shape of the as-prepared samples. The morphology of the nanoparticles of Ca_10−*x*_Ag_*x*_(PO_4_)_6_(OH)_2_, with 0.2 ≤ x ≤ 0.4 was investigated by SEM. In Figure 2 SEM image of Ca_10−*x*_Ag_*x*_(PO_4_)_6_(OH)_2_, with *x*_Ag_ = 0.2, *x*_Ag_ = 0.3, and *x*_Ag_ = 0.4 are shown. It can be seen that all the samples consist of elipsoidal particles. These results are well consistent with the TEM results, revealing that the doping components have little influence on the surface morphology of the samples. The spectrum and images confirmed the presence of silver on hydroxyapatite. The presence and uniform distribution of silver in the samples was confirmed. The EDAX spectrum of Ca_10−*x*_Ag_*x*_(PO_4_)_6_(OH)_2_ presented in Figure 3 confirmed the presence of calcium (Ca), phosphorus (P), oxygen (O) in all the samples. Elemental maps for Ca_10−*x*_Ag_*x*_(PO_4_)_6_(OH)_2_ with *x*_Ag_ = 0.4 are also shown in Figure 3. The EDAX spectrum of Ag:HAp confirms the presence of calcium (Ca), phosphor (P), oxygen (O), and silver (Ag) in the samples.

**Figure 4:**
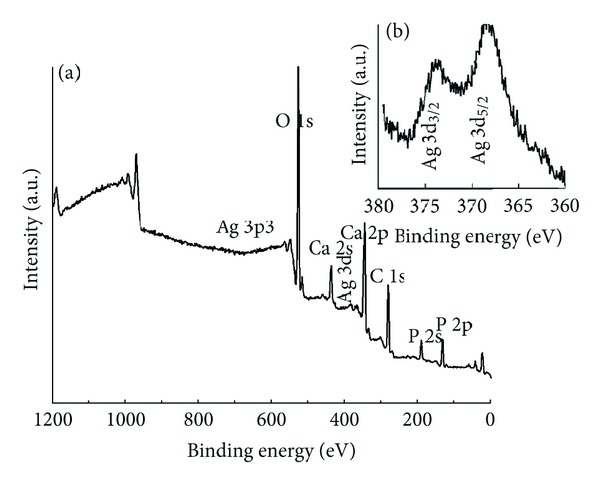
XPS general spectrum of Ca_10−*x*_Ag_*x*_(PO_4_)_6_(OH)_2_ powder with *x*_Ag_ = 0.4 (a) and narrow scan spectra of Ag element (b).

XPS technique has been used as a useful tool for qualitatively determination of surface components and composition of the samples.  Figure 4 shows the survey XPS narrow scan spectra of the as-prepared Ca_10−*x*_Ag_*x*_(PO_4_)_6_(OH)_2_ with *x*_Ag_ = 0.4 (a) and spectra of Ag element (b). In the XPS spectrum of Ag:HAp the binding energy of Ag (3d, 368.09 eV), Ca (2p, 347.3 eV), O (1s, 532.1 eV), and P (2p, 133.09 eV) can be obviously found. 

The peaks of Ag(Ag(3d_5/2_) 368.4 eV and Ag(3d_3/2_) 374.3 eV) agree well with the literature [17, 31]. XPS narrow scan spectra of Ag element is presented in Figure 4(b). XPS results provide the additional evidence for the successful doping of Ag^+^, in Ag:HAp. For XPS analysis the binding energies were calibrated with reference C 1s at 285 eV. By combination of XRD results, it can be concluded that these signals can be assigned to Ag:HAp nanopowder. XPS results provided the additional evidence for the successful doping of Ag^+^, in Ag:HAp. 

**Figure 5:**
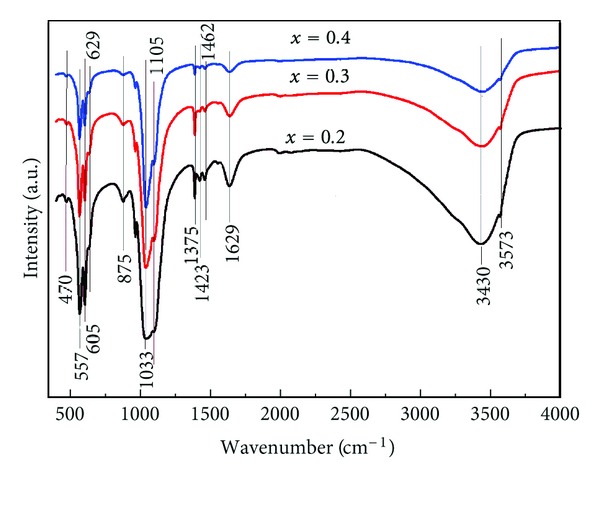
Transmittance infrared spectra of the Ca_10−*x*_Ag_*x*_(PO_4_)_6_(OH)_2_ samples with *x*_Ag_ = 0.2, *x*_Ag_ = 0.3 and *x*_Ag_ = 0.4.

FT-IR spectroscopy was performed to investigate the functional groups present in nano-hydroxyapatite, Ca_10−*x*_Ag_*x*_(PO_4_)_6_(OH)_2_, with *x* = 0.2, 0.3 and 0.4 obtained at 100°C by coprecipitation method (Figure 5). These date clearly revealed the presence of the various vibrational modes corresponding to phosphates and hydroxyl groups. The presence of strong OH^−^ vibration peak could be noticed for all the samples. The broad bands in the regions 1600–1700 cm^−1^ and 3200–3600 cm^−1^ correspond to H–O–H bands of water lattice [32–35].

 The large bands which were attributed to the adsorbed water diminished when *x*_Ag_ decreased. The changes are attributed to the substitution of Ag^+^ from Ca^2+^ into the apatite lattice. Bands characteristics to the phosphate and hydrogen phosphate groups in apatitic environment were observed: 557 cm^−1^, 629 cm^−1^, 605 cm^−1^, 960 cm^−1^, and 1000–1100 cm^−1^, for the PO_4_^3−^ groups [35, 36] and at 875 cm^−1^ for the HPO_4_^2−^ ions [37]. Moreover, it should be noted that the HPO_4_^2−^ band was present in all the spectra but the band diminished for high values of *x*_Ag_. The small CO_3_^2−^ band was presented in the spectra at 1375 cm^−1^ [37, 38].

However, the intensity of vibration peak decreases when *x*_Ag_ is 0.4. These results are in agreement with the X-ray diffraction patterns, evidencing the crystallized apatitic phase, which is the only one detected.

The emergence of bacterial resistance and multiresistance to antibiotics represents a major public health problem in Romania and also in the entire world, antibioresistance being declared by ECDC (European Center for Disease Control) as one of the major public health problems, besides HIV infection, tuberculosis, and influenza, rendering traditional antimicrobial treatment ineffective. The prognosis is worsened by the development of bacterial biofilms on tissues and biomaterials used in medicine due to their new behavior, called tolerance, defined as the ability to survive in the presence of bactericidal factors without necessarily expressing a genetic resistance [10, 11].

The biofilm phenotype can reduce antimicrobial susceptibility and increase tolerance up to 10 to 100, or even more, 1000 to 4000 times (impossibly to be used *in vivo*), evidently leading to clinical treatment failures of normal therapeutically doses of antibiotics. As a result, there is an increasing need to design new antibacterial and antifungal agents with better activity profiles and lower toxicity.

The qualitative screening of the antimicrobial activity of the tested compounds performed by using stock solutions of 5 mg/mL obtained in dimethylsulfoxide (DMSO) allowed the selection of the active compounds, indicated by the occurrence of growth inhibition zones around the spotted compound, with higher diameters than those obtained for the DMSO solvent. The tested compounds proved to be very active on *C. krusei* fungal strain, irrespective of the Ag concentration. 

None of the tested compounds was active against *P. aeruginosa*, *B. subtilis,* and *E. faecalis* strains. The specific antimicrobial activity revealed by the qualitative assay demonstrates that our compounds are interacting differently with the microbial targets, probably due to the differences in the microbial wall structures. The first step in the microbial cell-nanoparticle interaction is to attach and anchor to the surface of the cell wall, involving both electrostatic forces and molecular interactions. The Gram-negative bacteria possess an outer membrane with pores and a unique periplasmic space which is not found in Gram-positive bacteria, favoring the internalization of nanoparticles and their intracellular accumulation. The tested metal nanoparticles seemed to exhibit a better bactericidal effect on the Gram-negative strains, compared with the Gram-positive ones, accounting for the hypothesis that they cause changings in the bacterial membrane permeability, affecting the physiology and finally, the cell viability [39]. The interaction with the bacterial wall is the most probable cause for cellular death, taking into account that the size of the synthesized nanoparticles (5–30 nm) is surpassing the largest reported pore diameter in *E. coli*, with a pore hole diameter that may be up to 6 nm [13]. Therefore, the nanoparticles may be excluded from the cell interior according to the barrier properties of the cell envelope [40].

**Figure 6:**
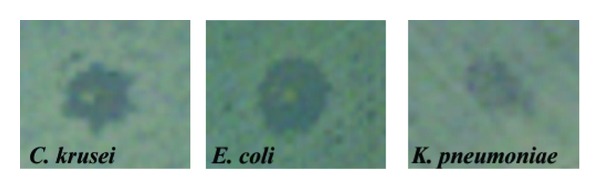
Qualitative assay of the inhibitory activity of Ag:HAp (*x*_Ag_ = 0.2) on *C. krusei* 963, *E. coli* ATCC 25922 and *K. pneumoniae* 2968 growth.

**Figure 7:**
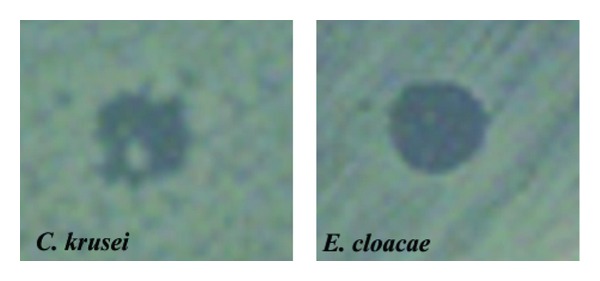
Qualitative assay of the inhibitory activity of Ag:HAp (*x*_Ag_ = 0.3) on *C. krusei* 963 and *E. cloacae* 61R growth.

**Figure 8:**
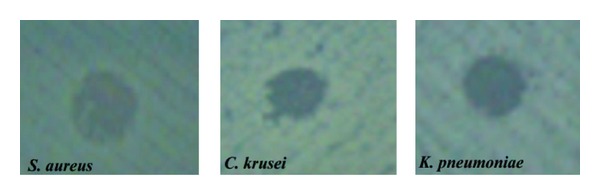
Qualitative assay of the inhibitory activity of Ag:HAp (*x*_Ag_ = 0.4) on *S. aureus* 0364, *C. krusei* 963, and *K. pneumoniae* 2968 growth.

The bactericidal effect of Ag:HAp (*x*_Ag_ = 0.2, *x*_Ag_ = 0.3, *x*_Ag_ = 0.4) in the culture media was studied by an adapted disc diffusion method on five different-strains (*E. coli* ATCC 25922, *K. pneumoniae* 2968, *E. cloacae* 61R, *S. aureus* 0364, *C. krusei* 963). The antibacterial activity of the tested compounds due to the presence of silver ions in the hydroxyapatite structure is well demonstrated by the considerable inhibition zones obtained against the *S. aureus* 0364, *C. krusei* 963, *K. pneumoniae* 2968, and *E. cloacae* 61R strains (Figures 6, 7, and 8). These strains were further selected for the quantitative assays of the MIC value.

It is also to be mentioned that DMSO did not exhibit any traceable antimicrobial activity at the studied concentrations, thus the solvent did not influence the biological activity of the tested substances. The inert substrata including the prosthetic medical devices represent risk factors for the occurrence of biofilm associated infections. Taking into account the differences in physiology and susceptibility to antibiotics of biofilm-embedded bacteria [41, 42] the compounds active against different bacterial strains in planktonic, free state were also investigated for their efficiency against the adherent cells grown in biofilms developed in plastic wells.

**Figure 9:**
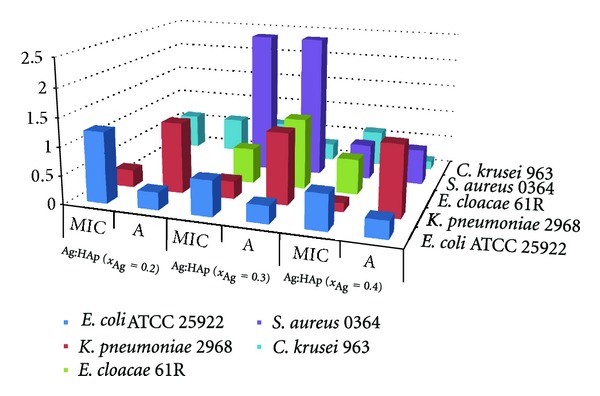
Graphic representation of the minimal inhibitory concentrations (MIC) and biofilm active concentration (*A*) of the selected compounds on the microbial strains susceptible to the compounds in the qualitative screening assay.

For the quantitative assays, the active compounds have been tested only on the strains which proved to be sensitive in the qualitative assays, showing growth inhibition zones (Figure 9). The experimental model uses mini volumes and multiple plastic well plates, allowing the simultaneous testing of a large spectrum of concentrations. Our quantitative assays proved that the tested compounds exhibited different activities on the planktonic and adherent cells.

The MIC of planktonic bacterial cells growth was higher than the initial biofilm active concentration in the case of *E. coli*, equal in the case of *E. cloacae* 61R, *S. aureus* 0364, and *C. krusei* 963 and lower in the case of *K. pneumoniae* 2968, for Ag:HAp (*x*_Ag_ = 0.2). For Ag:HAp (*x*_Ag_ = 0.3), the MIC of planktonic bacterial cells growth was lower than the initial biofilm active concentration in the case of *E. cloacae* and *K. pneumoniae* strains, equal in the case of *S. aureus,* and higher in the case of *E. coli* and *C. krusei*. 

**Figure 10:**
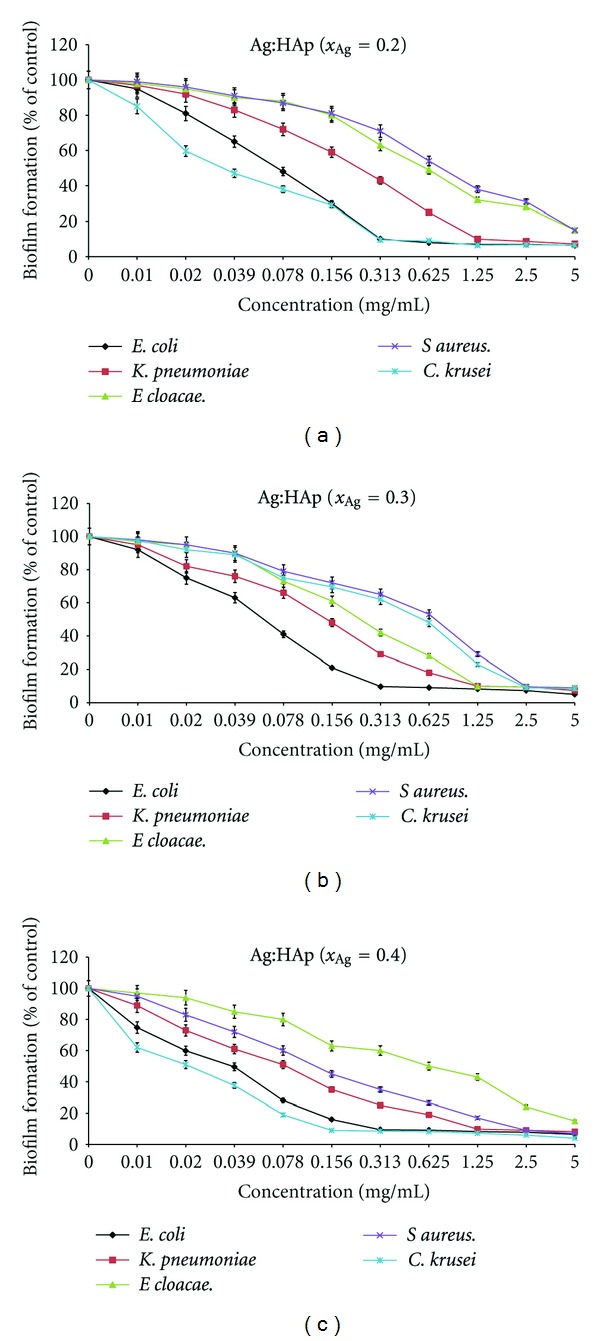
The inhibitory effect of Ag:HAp on the initial biofilm formation by *E. coli* ATCC 25922, *K. pneumoniae* 2968, *E. cloacae* 61R, *S. aureus* 0364, and *C. krusei* 963 strains.

**Figure 11:**
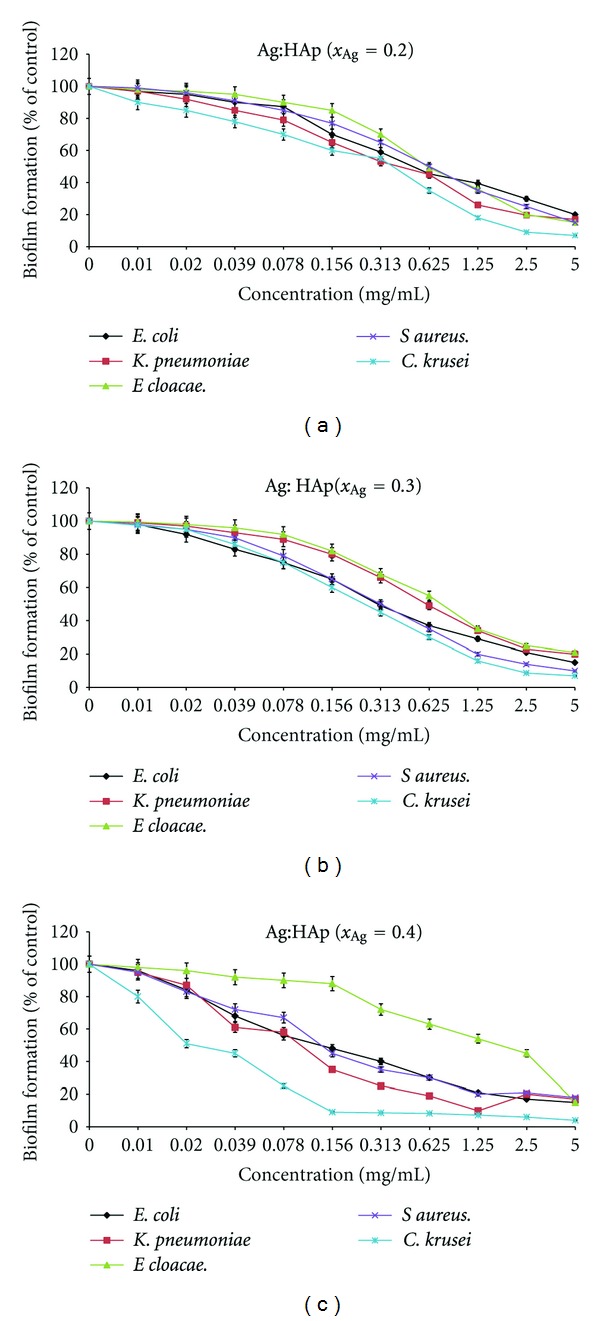
The inhibitory effect of Ag:HAp (*x*_Ag_ = 0.2, *x*_Ag_ = 0.3, *x*_Ag_ = 0.4) on preformed *E. coli* ATCC 25922, *K. pneumoniae* 2968, *E. cloacae* 61R, *S. aureus* 0364, and *C. krusei* 963 biofilms.

We have tested the obtained compounds for their influence both on the first step of bacterial adherence to the inert substratum and on 24 hours preformed biofilms (Figures 10 and 11). 

The results of the influence of Ag:HAp (*x*_Ag_ = 0.2, *x*_Ag_ = 0.3, *x*_Ag_ = 0.4) on biofilm formation by *E. coli* ATCC 25922, *K. pneumoniae* 2968, *E. cloacae* 61R, *S. aureus* 0364, and *C. krusei* 963 are shown in Figure 10. The inhibitory effect on the biofilm formation was dependent on the concentration of the tested compounds. It can be seen that Ag:HAp with *x*_Ag_ = 0.2, *x*_Ag_ = 0.3, *x*_Ag_ = 0.4 inhibited in a similar manner the biofilm formation by *E. coli* ATCC 25922 and *K. pneumoniae* 2968. On the other hand, the inhibitory effect on the preformed biofilms by *E. coli* ATCC 25922, *K. pneumoniae* 2968, and *E. cloacae* 61R was similar for the tested Ag:HAp samples (Figure 11). Biofilm active concentrations of the selected compounds on the microbial strains susceptible to the compounds in the qualitative screening assay are presented in Table 1. 

These results suggest that Ag:HAp could inhibit the early biofilm formation in a more important measure than the reduction of mature biofilm. Silver is known to inhibit the adherence of various microbial strains which can invade and grow within other organisms, causing diseases that can kill humans, animals, and plants. Furthermore, it is known that silver is one of the most nontoxic and safe antibacterial agent in medical products and consumables such as cosmetics and textiles. On the other hand, when the effective concentrations of silver nanoparticles on various types of organisms are determined, the silver nanoparticles can be well applied in therapy safely. However, metallic silver is not strong enough to be a load bearing metal implant. Therefore, silver doped hydroxyapatite might be useful in preventing postoperative infections. 

Several other studies demonstrated that the silver nanoparticles show the efficient antibacterial activity against *E. coli* and *S. aureus* [43–45]. Besides, a high concentration of silver nanoparticles may cause adverse health effects. For reducing the toxic effects of silver several biodegradable polymers were used for coating the silver nanoparticles. The recent studies on Ag:HAp nanopowders [17] obtained by coprecipitation method demonstrated a good antibacterial activity. Novel nanopowders based on silver doped hydroxyapatite will diminish adverse effects of the silver. The present study was also focused on the development of a biomaterial based on silver doped hydroxyapatite exhibiting antibacterial and antifungal properties, as demonstrated by our microbiological assays. These results are encouraging us to consider that nanosilver doped hydroxyapatite could be used for different biomedical applications, such as the treatment of wounds and burns infections, the wound dressings and coating of bone prostheses.

## 4. Conclusions

Coprecipitation method for synthesized the Ag:HAp using inexpensive and nontoxic compounds was summarized in this paper. On the other hand, the experimental conditions were found to control the morphology and size of the Ag-HAp particles when Ag concentration increases. The XRD studies have shown that the characteristic peaks of hydroxyapatite are present in each sample. No other phases were detected. The EDX, and XPS spectra confirmed the presence of silver in the samples. Additionally, the antimicrobial activity of the Ag:HAp nanopowder was assessed. The results obtained in this study demonstrated that silver doped hydroxyapatite nanoparticles may offer an effective alternative to antibiotic treatments, exhibiting a specific spectrum of antimicrobial activity, and also inhibiting the initial step of microbial biofilm development, but not the preformed biofilms, which proved to be more resistant and difficult to eradicate. 

## Figures and Tables

**Table 1:** Biofilm active concentrations of the selected compounds on the microbial strains susceptible to the compounds in the qualitative screening assay.

Ag:HAp	*x*_Ag_ = 0.2		*x*_Ag_ = 0.3		*x*_Ag_ = 0.4	
Tested parameter	Biofilm active concentration (mg/mL)		Biofilm active concentration (mg/mL)		Biofilm active concentration (mg/mL)	
	Initial biofilm	Preformed biofilm	Initial biofilm	Preformed biofilm	Initial biofilm	Preformed biofilm
*E. coli *	0.313	>5	0.313	>5	0.313	>5
*K. pneumoniae *	1.25	>5	1.25	>5	1.25	>5
*E. cloacae *	>5	>5	1.25	>5	>5	>5
*S. aureus *	>5	>5	2.5	>5	0.625	2.5
*C. krusei *	0.625	2.5	0.313	1.25	0.156	0.156
